# The MyRelief Digital Educational Self-Management Program for Persistent Low Back Pain: Feasibility Uncontrolled Trial

**DOI:** 10.2196/74948

**Published:** 2025-08-27

**Authors:** Caroline Larsson, Joanne Marley, Flavia Piccinini, Sarah Howes, Elisa Casoni, Vincenzo Aschettino, Carlos Vaz de Carvalho, Suzanne McDonough

**Affiliations:** 1 Department of Health Sciences, Lund University, Sölvegatan 19, Lund, 223 62, Sweden, 46 736673026; 2 School of Health Sciences, Ulster University, Belfast, United Kingdom; 3 IRCCS INRCA - Istituto Nazionale Ricovero e Cura Anziani (National Institute of Health and Science on Ageing), Ancona, Italy; 4 School of Health Sciences, Ulster University, Derry-Londonderry, United Kingdom; 5 Tech4Care srl, Ancona, Italy; 6 Instituto Superior de Engenharia do Porto, Porto, Portugal; 7 RCSI University of Medicine and Health Sciences, Dublin, Ireland

**Keywords:** low back pain, feasibility, self-management, online education, mooc, massive open online course

## Abstract

**Background:**

Low back pain (LBP) is a leading cause of work absence globally. Digital interventions have the potential to increase access to self-management support for individuals with persistent LBP.

**Objective:**

This study aims to evaluate the feasibility, usability, and acceptability of a digital educational program (MyRelief) designed to support self-management strategies for people with persistent LBP.

**Methods:**

A prospective uncontrolled feasibility study was conducted across 4 countries (Italy, Portugal, Sweden, and the United Kingdom) between 2020 and 2021. Adults in employment with nonspecific persistent LBP (>3 mo) with access to the internet were eligible to participate. Participants were given access to MyRelief, an 8-unit evidence-based educational self-management program. The feasibility of the MyRelief program was assessed using recruitment rates, an a priori success threshold of >70% of the target sample (50 participants), and a retention <35% dropout rate. Pre- and postintervention measures of functional disability were assessed using the Oswestry Disability Index (ODI), and health-related quality of life using the 5-level EuroQol questionnaire. Additional postintervention measures included the Patient Enablement Instrument and the System Usability Scale. Quantitative data were analyzed descriptively, and qualitative feedback was analyzed using a reflexive analytical approach.

**Results:**

The recruitment feasibility threshold was met, and 40/50 (80%) participants (19 male and 21 female; mean age 57 years) were enrolled in the study. A total of 17 participants (11 male and 6 female) completed both the baseline and 12-week follow-up questionnaires. This represented a retention rate of 42.5% (17/40) and a dropout rate of 57.5%, which did not meet the a priori criteria of <35% dropouts. Approximately half of the participants presented with low baseline disability scores (mean ODI 24.0; 95% CI 18‐31) with no significant change at follow-up (mean ODI 23.9; 95% CI 16‐31). The 5-level EuroQol questionnaire scores improved from 0.68 (95% CI 0.608‐0.76) to 0.72 (95% CI 0.66‐0.79), indicating a clinically significant change. Patient Enablement Instrument scores postintervention were high (mean 5.31), indicating good perceived enablement. The mean System Usability Scale score was 72.4 (95% CI 67.5‐73.3), indicating a good level of perceived ease-of-use. Overall, the quality of outcome measure completion was high (100%). Qualitative feedback indicated areas for improvement relating to challenges around access and navigation within the website.

**Conclusions:**

The MyRelief study demonstrated feasibility in terms of recruitment but not retention. However, low baseline disability levels are not representative of the wider persistent LBP population. Future studies should broaden recruitment strategies, in particular, by recruiting from health care settings to improve representativeness. Although usability met industry standards, qualitative feedback suggests that navigation and accessibility require further optimization to better align with end user preferences for digital health interventions.

## Introduction

### Overview

Persistent low back pain (LBP) is among the most prevalent, disabling, and expensive conditions to treat [1]. The 2023 Global Burden of Disease study estimates that 619 million people lived with LBP in 2020, with numbers projected to rise to 843 million by 2050—nearly 39% of the associated disability can be attributed to occupational ergonomic exposures, smoking, and high BMI [2]. It can affect an individual’s ability to work, maintain relationships, and function in normal day-to-day life [1]. Recent European data confirm LBP’s labor-market impact: in a nationwide Swedish cohort of more than 830,000 workers, any episode of LBP-related sickness absence tripled the likelihood of subsequent disability retirement (hazard ratio 3.2) [3]. The UK evidence from the Whitehall II data similarly reports that recurrent back pain is associated with higher odds of exiting from paid work for health reasons, particularly in those in lower and middle grade jobs [3].

As the incidence of LBP is becoming more prevalent globally, with the steepest relative increases projected for low- and middle-income countries [4], where resources may be limited, it is important that accessible and effective interventions are available. The World Health Organization’s 2023 Digital Health Strategy cites musculoskeletal conditions as a priority area for scalable eHealth solutions in resource-constrained settings. Since most LBP is not considered to be related to a specific identifiable spinal pathology, digital educational self-management programs are appealing as they encourage and facilitate wider participation. Digital delivery, including web-based and mobile formats, expands reach, allows asynchronous access, and can reduce per-capita costs by leveraging economies of scale [5].

Digital educational self-management programs have demonstrated effectiveness in a range of chronic disorders such as cardiovascular disease [4], chronic obstructive pulmonary disease [6], and diabetes [5]. For LBP specifically, a 2025 systematic review of digital interventions for employed adults with LBP identified small-to-moderate improvements in pain, disability, and work ability across 18 trials, although study quality was mixed and attrition was high [7]. The following are the findings of 2 recent randomized controlled trials: the UK SupportBack 2 trial (n=1201) found that an internet-delivered program with or without brief clinician support was safe, cost-effective, and produced modest reductions in disability at 12 months [8]; the Scandinavian selfBACK trial (n=346) reported a statistically significant reduction in Roland-Morris disability scores that persisted to 9 months among workers with physically demanding jobs [9]. However, digital interventions to date appear to largely focus on individuals seeking care within health systems, limiting accessibility. An option for improving this and scalability is by using a “MOOC” (massive open online course) format. MOOCs are a type of digital course designed using principles of educational technology. For employees with persistent LBP, a MOOC offers flexible, asynchronous learning that can be fitted around working hours and accessed from any location with an internet connection. While MOOCs have shown promise during COVID in helping health workers through the provision of health education [9], the feasibility of using this format to facilitate an educational self-management program for working individuals with back pain has not been investigated.

The MyRelief MOOC was developed specifically to support individuals in employment with LBP. The materials were derived from a synthesis of consistent recommendations across 6 clinical guidelines [10-15]. The key focus of the MyRelief program was to provide a summary of evidence-based information in user-friendly formats.

The aim of this study was to determine the feasibility and acceptability of the MyRelief MOOC.

### Aim and Objectives

#### Aim

This study aimed to explore the feasibility, usability, and acceptability of a digital education program for people with persistent LBP.

#### Objectives

The objectives of the study are to evaluate the feasibility of the digital education program based on recruitment, retention, and completion of outcome measures; to assess the usability of the intervention using an industry standard tool to understand participants’ ease of interaction with the digital education program; and to evaluate the acceptability of the content and format of the digital education program through qualitative feedback.

## Methods

### Overview

The MyRelief study protocol, providing the rationale and description of the study, has been published elsewhere [16]. This paper was prepared in accordance with the Consolidated Standards of Reporting Trials (CONSORT) guidelines for reporting of a pilot or feasibility trial, and a CONSORT checklist has been included as Checklist 1.

### Study Design

The MyRelief study was a multicenter, multinational, uncontrolled feasibility study in Italy, Portugal, Sweden, and the United Kingdom. A fifth partner, Lithuania, contributed as a technology specialist but was not directly involved in the feasibility study. A separate paper was published [17] focusing on the technological aspects of the study. Ethics approval was obtained from the relevant ethics committee in each country. This study was funded by the European Commission’s Erasmus+ Program.

### Participants and Recruitment

Recruitment of adult workers with nonspecific, persistent LBP, present for at least 3 months, took place at the 4 study sites in Italy, Portugal, Sweden, and the United Kingdom between 2020 and 2021. Recruitment took place via several methods depending on each country's ethical approval, for example, via waiting list for outpatient rehabilitation services (Italy), email circulated to all staff and students at University (Sweden and the United Kingdom) or patients at a pain clinic (Portugal) or Social Media (Italy, Sweden, the United Kingdom, and Portugal). For the latter approach, a Facebook page was created with information about the project. Links to this page were posted on Instagram, Twitter, and other relevant forums. Individuals with unexplained symptoms, underlying pathology, or specific LBP were excluded, as were those who were not interested in using a digital intervention delivered via computer, laptop, tablet, or mobile. A full list of inclusion/exclusion criteria is in our study protocol [16].

Regardless of the method of recruitment, potential participants accessed the study information via a link provided in the recruitment material. If interested, they completed a web-based inclusion/exclusion questionnaire. Those meeting the inclusion criteria were given access to a digital consent form. After completing the consent form, they received access to the MyRelief study website to complete the baseline assessment and access the digital education program.

All participants were also asked to enter their email address and phone number to enable reminders and contacts with those interested in participating in the qualitative interview.

Various strategies to enhance recruitment were adopted in the different countries. In Sweden and the United Kingdom, interested participants responded via email to a member of the research team who checked eligibility and gave an overview of the study, answering any queries, prior to providing the study web link. The researcher arranged email, telephone, or video call support, depending on participant preference, to support them to get started with the system and checked in to resolve any technical issues. In Italy, multiple routes (email, telephone, and in-person recruitment meetings) were used to recruit participants, who were staff at the research hospital (nurses), local university (employees and administrative staff), and the general public. In Portugal, participants were recruited by direct contact with private clinics dealing with the issue.

### Intervention

The MyRelief digital education program was designed to be accessed on a computer or a mobile device. The MyRelief content was informed by focus groups with adult workers with persistent LBP, LBP guidelines, and the clinical expertise of the research team. It comprised 8 modules which covered the following topics: understanding LBP; physical activity and exercise in relation to LBP; psychological factors; sleep/nutrition; management of LBP in the workplace; communication with health care providers; and other issues related to LBP (eg, medication, scans, and alternative therapies). Each module was designed to be short enough to complete during a work break, that is, <20 minutes, and was made up of short written and video content, as well as knowledge quizzes. Participants received 4 weeks’ access to the digital education program prior to being invited to complete the postintervention assessment. Thus, completion of 2 online modules per week was encouraged to facilitate completion of the course within the timeframe.

### Feasibility

The feasibility of the study was assessed as the ability to recruit participants and retain them in the study, and completion of functional disability and quality of life measures. Recruitment was assessed as the proportion of the recruitment target met during the timeframe. A record was kept of the number of individuals who entered the system, completed the screening questions, and provided consent. Retention was assessed by calculating the proportion of participants who completed outcome assessments at both baseline and postintervention timepoints. Completion of outcome measures at baseline and postintervention timepoints was recorded.

### Usability

Participant views on usability and satisfaction with the system were assessed postintervention using the System Usability Scale (SUS), which is an industry standard measure with validity to measure a variety of aspects of usability, such as the need for support, training, and complexity [18].

### Acceptability

Qualitative feedback was assessed through semistructured telephone interviews with a subgroup of study participants (Italy) and comments provided through email correspondence between participants and the study teams (Sweden and the United Kingdom). Semistructured interview questions included feedback on participants’ experience using the digital education program, likes and dislikes, perceived usefulness, problems experienced, and suggestions for improvement.

### Patient Characteristics

Participant characteristics (demographics, history of LBP, type of occupation, etc) were collected at baseline via the study website. Type of occupation was defined as sedentary (mostly sitting, eg, office worker, driver, and professional), light manual (mostly standing or walking, eg, retail worker, health care worker, and cook), heavy manual (heavy labor, eg, farming, construction, and cleaning), unsure, or other.

The following outcome measures were collected at baseline and postintervention via the study website:

1. Functional disability was measured using the Oswestry disability questionnaire (ODQ) v2.1a, a valid and reliable measure of pain and physical function in people with LBP [19,20]. A minimum important change of between 10 and 12 points over time, or an improvement from baseline of between 20% and 30% for an individual, has been recommended [21].

2. Health-related quality of life was measured using the EuroQol questionnaire (EQ-5D-3L), which has been recommended for use in LBP research [22]. A minimum important change of 0.03 points for the categorical scales of the EQ-5D and 10.5 points for the EQ-5D Visual Analog Scale has been suggested [22].

The following outcomes were measured at postintervention only:

3. Change in understanding and coping with their LBP was assessed via the study website using the Patient Enablement Index (adapted to reflect enablement after engaging with the system), which is considered “the gold standard” for measuring enablement [23,24]. This measure is calculated as a sum score ranging between –6 to 10 points. A score greater than 6 points is suggested to reflect “high” enablement [25].

4. SUS: Scores above 70 can be interpreted as good usability, while scores below 50 indicate unacceptably low usability according to Bangor et al [18].

5. Semistructured interview (see above).

### Statistical Analysis

Statistical analysis was performed using Microsoft Excel. Participant characteristics and feasibility outcomes for recruitment and retention were presented as frequency and percentage (%), mean and SD, or median and IQR based on the distribution of the data.

The following feasibility criteria were applied: observed recruitment rate falls short of 70% of that anticipated, overall drop out of over 35%, no apparent change in the outcomes with confidence intervals that include large negative values, feedback from participants that they were unable to complete (or lack of engagement with) MyRelief, feedback from participants that suggests that the MyRelief content or format was not acceptable or usable.

Clinical and usability outcomes were reported descriptively as means and 95% CIs; significance tests were not performed in this feasibility study.

### Changes From Study Protocol

A number of pragmatic changes were implemented during study delivery. Initially, study recruitment was entirely online via a web link. Engagement was poor with this automated recruitment method, so additional methods were implemented to support recruitment. Changes related to researcher-supported recruitment are described above in the “Participants and Recruitment” section. In addition to these changes, participants also received emails (Sweden, the United Kingdom, and Portugal) or telephone reminders (Italy) to complete the follow-up questionnaire.

The study protocol outlined plans to collect qualitative data from participants related to usability and acceptability at all study sites; although 3 follow-up telephone interviews were successfully conducted in Italy, Sweden, and the United Kingdom, informal participant feedback was collated via telephone and email correspondence due to challenges with recruitment.

### Ethical Considerations

Ethics approval for this study has been obtained from the relevant ethics committee at each intervention site (Swedish Ethical Review Authority Dnr 2020-03536). The study was classified as human research involving minimal risk. The trial has been registered on ClinicalTrials.gov (NCT03575091). Regardless of the method of recruitment, potential participants accessed the study information via a link provided in the recruitment material. If interested, they completed a web-based inclusion/exclusion questionnaire. Those meeting the inclusion criteria were given access to a digital consent form. The consent covered both primary data collection and potential secondary analysis of deidentified data. Participants were informed of their right to withdraw from the study at any time without any consequences. All data were deidentified prior to analysis, and all materials are presented to ensure complete anonymity of the study participants. Participants were assigned unique study codes, and no personally identifiable information was stored with the research data. All digital files were stored on secure, password-protected servers in accordance with institutional data protection guidelines. Participants did not receive any compensation for their contribution to the study.

## Results

### Participant Baseline Characteristics

A total of 40 participants (19 male and 21 female; mean age 57 years) were recruited. Baseline demographics are reported in Table 1. Mean pain duration was 11.5 years and differed between countries from 7 years (Portugal) to 16.3 years (Sweden), and among individuals, it ranged from 1 year to 38 years. A total of 28 (70%) of participants reported a sedentary occupation. Among the participants in Portugal and Italy, these levels were lower, 40% and 54%, respectively (Table 1).

**Table 1.** Baseline demographics of participants by country and in total in the MyRelief feasibility study of a digital self-management program for persistent low back pain. The study included participants recruited from multiple European countries.

	Italy (n=13)	Northern Ireland (n=13)	Portugal (n=5)	Sweden (n=9)	Total (n=40)
Age (years)					
Mean (SD)	57.2 (2.8)	56.3 (2.0)	59.8 (3.4)	57.4 (7.7)	57.3 (4.2)
Range	55‐65	55‐61	55‐64	48‐76	48‐76
Sex, n (%)					
Female	8 (62)	6 (46)	0 (0)	7 (78)	21 (53)
Male	5 (38)	7 (54)	5 (100)	2 (22)	19 (47)
Pain duration in years					
Mean (SD)	12.6 (10.4)	8.8 (9.5)	7.0 (3.9)	16.3 (13.3)	11.5 (10.5)
Range	2‐30	1‐30	1‐11	2‐38	1‐38
Occupation category, n (%)					
Sedentary	7 (54)	11 (85)	2 (40)	8 (89)	28 (70)
Light manual	6 (46)	2 (15)	2 (40)	0 (0)	10 (25)
Heavy manual	0 (0)	0 (0)	1 (20)	1 (11)	2 (5)

### Feasibility

#### Recruitment and Retention

**Figure 1.**
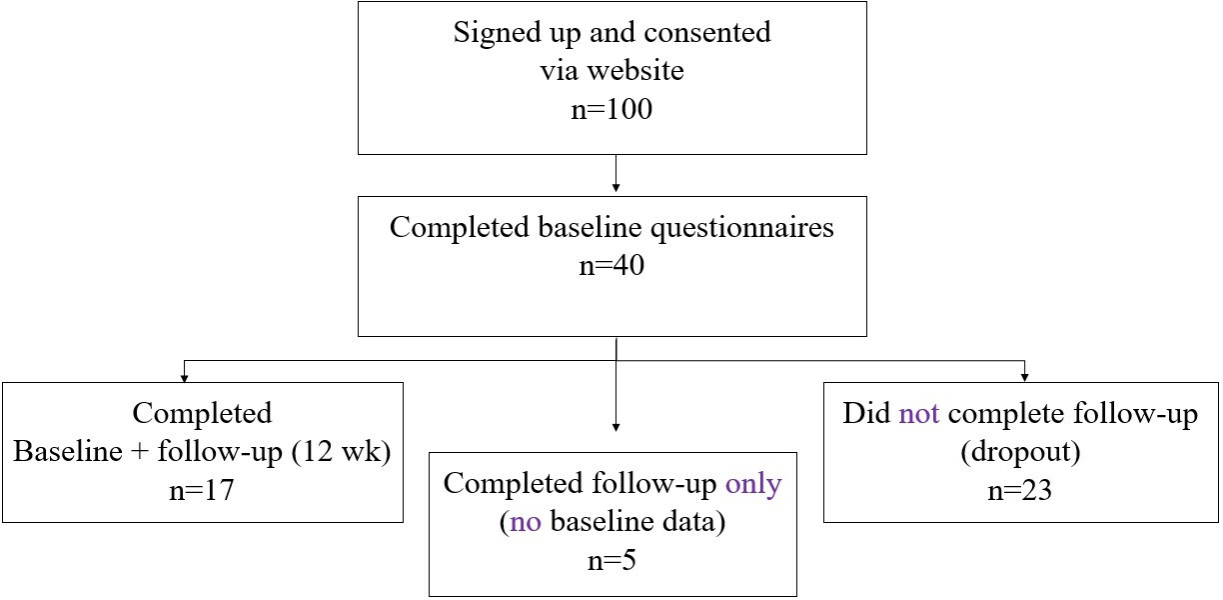
Participant flow diagram for the MyRelief digital self-management program feasibility study. Out of 100 individuals who signed up and gave consent via the study website, 40 participants completed the baseline questionnaire. Of these, 17 completed both baseline and 12-week follow-up assessments, while 23 did not complete follow-up (dropouts). An additional 5 participants completed only the follow-up questionnaire.

The flow diagram in Figure 1 shows the participant flow in the study. A total of 100 individuals (44 male and 56 female) created a MyRelief account and reached the study landing page. Creating an account generated a system “login” but did not in itself constitute study enrollment. Of these 100 account holders, 40 completed the electronic consent form and baseline questionnaires and were therefore classified as recruited participants. Our a priori feasibility target was to enroll 50 participants [16]; we therefore achieved 80% (40/50) of the planned sample size.

Recruitment and retention follow-up questionnaires were completed by 17 (11 male and 6 female) of the 40 recruited participants, yielding a retention rate of 42.5% and a corresponding dropout rate of 57.5%. Retention is expressed relative to the 40 recruited participants. However, an additional 5 people completed only the follow-up questionnaire, increasing the total follow-up responses to 22.

#### Completion of Outcome Measures

Quality of outcome measure completion was good, with valid and usable data obtained for all clinical outcomes from all participants (n=17). The outcome measures are described in Table 2.

**Table 2.** Outcome measures for participants (n=17) with complete data at both baseline and 12-week follow-up in a digital self-management program for persistent low back pain.

	Baseline	Follow-up
ODQ^a^, mean (95% CI)	24.0 (18-31)	23.9 (16-31)
ODQ categories, n (%)		
>21 (moderate)	8 (47.0)	7 (41.2)
<21 (low)	9 (53.0)	10 (58.8)
Pain intensity, mean (95% CI)^b^	1.58 (1.07‐2.10)	1.50 (1.07‐1.93)
EQ-5D-3L, mean (95% CI)^c^	0.678 (0.598‐0.759)	0.723 (0.662‐0.785)
EuroQol Visual Analog Scale, mean (95% CI)^c^	67.76 (60.71‐74.82)	70.59 (65.14‐76.03)

a ODQ: Oswestry disability questionnaire (0‐100), a higher score indicates more disability. A minimal clinically important difference between 10 points over time, or an improvement from baseline of between 20% and 30% for an individual has been recommended.

b Pain intensity: responds to the first item of ODQ “What is your pain intensity at the moment,” scores ranging between 0 (no pain at the moment) to 5 (the pain is the worst imaginable at the moment).

c EuroQol questionnaire (EQ-5D-3L) Weighted Health Index, TTO (–0.59 to 1.0) a higher score indicates better quality of life. A change of 0.03 points for the categorical scales of the EQ-5D-3L and 10.5 points for the EuroQol Visual Analog Scale can be categorized as clinical important.

**Figure 2.**
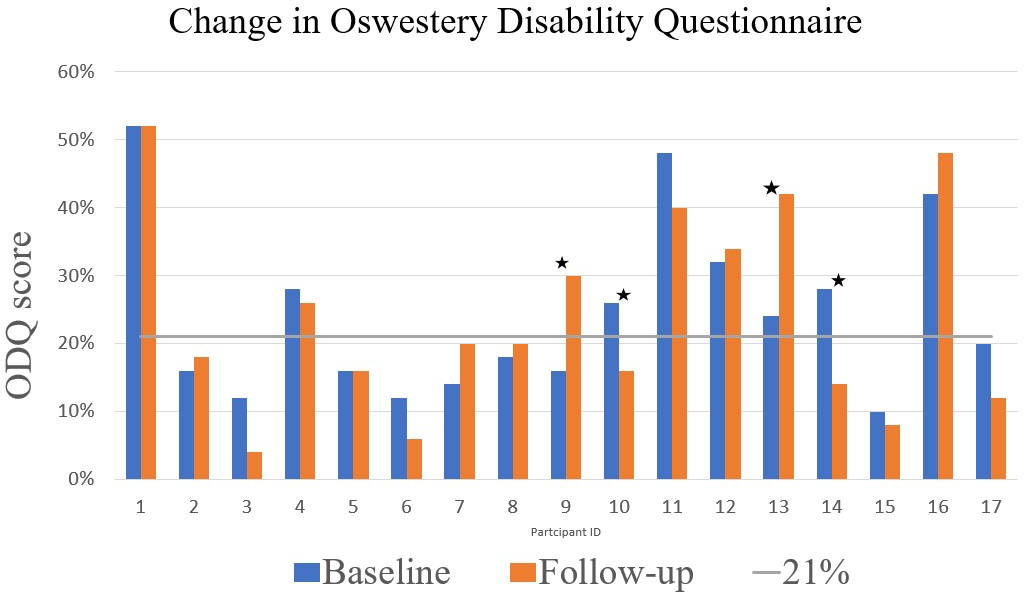
Change in ODQ scores from baseline to 12-week follow-up in participants taking part in a digital education program for persistent low back pain (n=17). Scores below and above the gray line represent mild and moderate disability, respectively. *Indicates a change >10 points, which represents the minimal clinically important difference (MCID) for ODQ. ODQ: Oswestry Disability Questionnaire.

Across all participants, the mean baseline ODQ was 24.0 (95% CI 18‐31), which would correspond to moderate disability [19,20]. Mean pain intensity at baseline was 1.58 out of 5 and ranged between 0 and 2, except for one participant who had scored 4. The mean pain intensity showed a positive correlation to the total mean ODQ (*r*=0.71). No change was found for the total ODQ mean at follow-up (23.9, 95% CI 16‐31). However, 4 individual participants achieved change between baseline and follow-up corresponding to the minimal clinically important difference of the ODQ (a score >10%), where 2 participants improved from moderate to low disability, one participant deteriorated from low to moderate disability, and one individual showed an increase in disability but remained within the range of moderate disability (Figure 2).

A clinically important increase was found for the mean EQ-5D Weighted Health Index between baseline (0.678, 95% CI 0.598‐0.759) and follow-up (0.723, 95% CI 0.662‐0.785). The EQ-5D visual analog scale also showed an increase from 67.76 (95% CI 60.71‐74.82) to 70.59 (95% CI 65.14‐76.03); this was not a clinically important change. At the individual level, the majority of participants increased or maintained their EQ-5D scores at follow-up (Table 2).

A total mean for the Patient Enablement Index was calculated as 5.31 (95% CI 3.94‐6.70) at follow-up. The median value was 5.5 points. A Patient Enablement Instrument (PEI) greater than 6 points is suggested to reflect “high” enablement [25]. Two participants (9%) reached the highest score (12 points), and 2 participants (9%) scored the lowest score (1 point). Thus, no floor or ceiling effects were found for the total score.

### Usability

#### Overview

**Figure 3.**
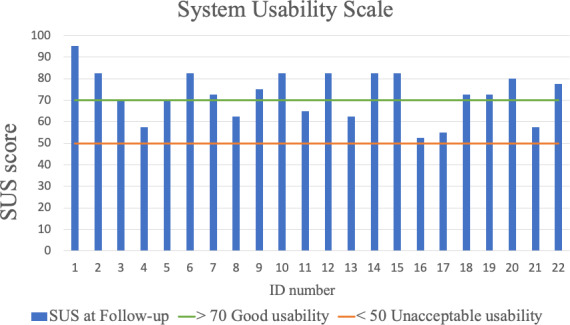
Participant scores of the SUS at 12-week follow-up in participants taking part in a digital education program for persistent low back pain (n=22). A higher score indicates better usability. Scores above 70 indicate good usability, while scores below 50 indicate unacceptably low usability. SUS: System Usability Scale.

A total of 22 participants responded to the SUS. The total mean SUS score was 72.4 (95% CI 67.5‐73.3), which would be interpreted as good [18]. The median score was 75. The score covered the range from 52.5 (1 person) to 95.0 (1 person), with 13 out of 22 participants scoring above 70. Figure 3 describes the participant scores of SUS. Qualitative feedback confirmed some challenges with usability in terms of access to and navigation within the website (Textbox 1).

Textbox 1. **Qualitative feedback on the acceptability of the MyRelief educational web page. Qualitative feedback was gathered via interviews (Italy) and email (Sweden and the United Kingdom), focusing on participants’ experiences with the digital education program.**

Pros

The international experts together. The fact of having input from experts from different countries was very good.

It was interesting, I really enjoyed it.

There were things I did not know that came in handy. Like the talk about stress can lead to spinal problems. It was very useful.

Cons

Access was a bit complicated at first, but once understood, everything went well.

Perhaps I expected a little more from the ergonomics point of view. Ergonomics applied to everyday life (eg, posture in the workplace).

The exercise program was very difficult. The exercises are tiring and difficult.

A slightly more graduated/modulated exercise program would be needed.

It is informative but not sufficient to support a change in lifestyle.

It lacks the possibility of face-to-face interaction and should be integrated with other interventions in order to work.

Not all speakers had the same communication skills.

All physiotherapists were female, while the doctor was male!

Unfortunately, some of the lessons were not in Italian, I had difficulties.

Difficult to navigate at the web page.

Some quiz questions were perceived as provocative.

Difficult to access the web page.

#### Acceptability

Qualitative feedback also made suggestions on the content of the website that were both positive and negative, for example, “having input from experts from different countries was very good“ and “the exercise programme was very difficult” (Textbox 1).

## Discussion

### Principal Findings

This study explored the feasibility, usability, and acceptability of a digital education program for people living and working with persistent LBP. Recruitment of study participants reached the a priori feasibility criteria (>70% recruitment). However, retention was challenging, and the a priori feasibility criteria were not met (<35% dropouts). The quality of outcome measure completion was good. The SUS indicates that the MyRelief website met minimal industry standards, but qualitative feedback confirmed some challenges with usability in terms of access to and navigation within the website. Although mean functional disability was moderate, actually, half of our sample only had mild functional disability and low pain intensity. A clinically important increase between baseline and follow-up was found for EQ-5D Weighted Health Index, suggesting a meaningful improvement in patients’ perceived health status. This aligns with the idea that participation in patient education sessions may play a positive role in enhancing quality of life. While the observed improvement is promising, further exploration is needed to determine whether this alone is sufficient to sustain long-term improvements in quality of life.

### Feasibility

#### Recruitment and Retention

While the idea of an open, globally accessible, evidence-based self-management intervention for LBP is appealing, this multi-country study also revealed several challenges. Although we met our a priori recruitment target of 50 participants, it is worth noting that despite 100 individuals signing up and providing consent to participate, only 40 completed the baseline questionnaire, corresponding to a 40% response rate among those registered.

Based on the a priori recruitment target of 50 completed baseline assessments, the study achieved 80% of its goal, thereby meeting the predefined feasibility threshold (>70%).

Once recruited, we encountered a second significant challenge in terms of retention of the participants who have signed into the study website, as retention at follow-up was more limited: 17 participants completed both baseline and follow-up assessments, yielding a retention rate of 42.5% and a dropout rate of 57.5%, which did not meet the preset criterion (<35% dropouts). These difficulties reflect a broader challenge in trial recruitment, as shown in a comprehensive review of 151 randomized controlled trials, where only 56% met their final recruitment target, highlighting the ongoing complexity in recruiting for randomized controlled trials and the need for more effective planning [26]. It could also be postulated that using a completely web-based recruitment strategy is not advisable. A recent meta-analysis found web-based recruitment strategies superior to offline recruitment initiatives when measuring recruitment effectiveness by recruitment rate and cost-effectiveness. However, this study found that offline recruitment outperforms web-based recruitment when converting potential participants to actual enrollees [27]. Engaging with participants through screening and providing an opportunity for discussion appeared to enhance recruitment in our study. These findings highlight important challenges in sustaining engagement in fully digital, self-directed interventions. Factors such as lack of personal contact, time constraints, or limited perceived relevance may have contributed to lower retention. Future studies may benefit from incorporating hybrid delivery models, tailored reminders, or additional onboarding support to improve participant engagement.

#### Completion of Outcome Measures

The baseline mean ODQ score of 24.0 (95% CI 18‐31) was notably lower than in comparable studies examining disability levels among individuals with persistent LBP recruited via health care practitioners [28,29] but similar to studies, like ours, that recruited directly from the community [30]. This may explain in part the marginal improvements we observed in terms of numbers meeting the minimal important change criteria [21]. These findings suggest that the cohort under study might not be the most responsive to the intervention being evaluated. It is likely that a digital program like MyRelief could have a greater impact on individuals with more severe LBP. Given these insights, it seems prudent to target patients awaiting care in primary health care settings, as they may represent a more suitable demographic for intervention. Consequently, future recruitment efforts could be optimized by engaging health care providers in the process.

The mean score for patient enablement reported was 5.31, a level that corresponds to previously published studies reporting on patient enablement [31-33]. Patient enablement emphasizes patients’ involvement in their care, understanding of their health, and participation in decision-making. The original PEI assesses change in patients’ ability to cope and their understanding of their disease, following a consultation [34]. We used a modified PEI to assess the impact of MyRelief on knowledge acquisition for self-management strategies. Even though our study’s relatively high enablement level may stem from MyRelief’s focus on knowledge acquisition rather than solving complex issues typical of health care consultations. Our findings suggest that MyRelief has the potential to influence patient enablement, although the specific components contributing to this improvement require additional exploration.

### Usability

The SUS score of 72.4 suggests that our digital education program meets acceptable usability standards and can be considered to offer good usability [18], indicating that MyRelief digital education has the prerequisites required for further development. In comparison to other digital interventions to support and facilitate self-management of LBP, previous mean scores have ranged from 64.7 to 70.5 [17,35]. However, comparability is limited as previous studies have mostly focused on the usability of mobile applications. Nonetheless, it is promising that the current digital program was perceived as usable by MyRelief study participants. The current platform did not support detailed use analytics, which limited our ability to assess which modules were most engaging or where participants may have disengaged. Future versions should include integrated tracking of module completion, time spent, and quiz interaction to better understand user patterns and adapt content accordingly. There was a lack of qualitative feedback on usability in our study, but the feedback that was collected primarily highlighted issues around accessibility to the website. It has been reported that a clear and intuitive navigation system can significantly enhance accessibility, ensuring easy access to important information [36]. Improved understanding regarding accessibility and usability preferences from the end users’ perspective is required in order to improve the utility of the digital education format for health-related programs.

### Acceptability

Also, the acceptability of the MyRelief digital education program was assessed through qualitative feedback collected via semistructured interviews and email correspondence. Participants generally appreciated the relevance of the content and its focus on self-management, with several highlighting the usefulness of practical examples. However, feedback also revealed that some participants found the material too basic in relation to their existing knowledge or needs. This suggests that although the education program was acceptable to many, others desired greater depth or personalization.

While the overall design and delivery format were well received, some users expressed preferences for shorter modules or more interactive elements. These comments provide important insights for future refinement of the education program. Acceptability could potentially be improved by tailoring content to different levels of prior knowledge or by offering optional advanced sections. Although qualitative feedback was limited in volume, it was important in identifying strengths and specific areas for improvement.

### Strengths and Limitations

We conducted this study in accordance with CONSORT guidelines and followed a prospectively registered protocol [16]. A key strength and a novelty of this trial is the transnational approach in developing evidence-based material that could facilitate health education, providing an infrastructure that can be upscaled to reach more learners, leveraging digital tools and materials. However, the transnational development process also revealed that the cultural context of health differed between countries. A lot of the workload within the project was attributed to developing materials that required multiple translations and heavy editing to ensure the advice and information were appropriate for and sensitive to different cultural contexts. To maximize the utility of interventions delivered using a digital education format, cultural contexts of health need to be considered. The multi-country nature of this study revealed valuable insights into the importance of cultural and contextual adaptation. Although no formal subgroup comparisons were made, observations from the project teams indicated differences in how participants across sites perceived the program’s content and usability. Some adjustments—such as modifying terminology, examples, and health care system references—were made during translation to improve relevance. Nonetheless, variations in language availability and local health literacy may have influenced both engagement and acceptability, highlighting the need for cocreation [37-39] and iterative user testing in future implementations. Although we conducted initial focus groups with individuals with LBP, further iterative review opportunities with patient representatives and clinicians were not feasible within the project timeline. For future studies, more focus should be dedicated to structured cocreation and the involvement of health care providers in this process, including repeated feedback loops from patients in each participating country. In addition, the diversity of learners’ own goals, motivations, and interests needs to be satisfied through the design, as the learning experience will benefit from personalization [40]. Another key consideration for future development is the importance of using an administratively simple and flexible course system. The current platform lacked integrated tracking features, which limited our ability to monitor progress and engagement. To minimize challenges with technology, interdisciplinary collaboration with developers and clinician academics is crucial. For digital education programs to be scalable and sustainable, they must also be supported by systems that allow easy management of participants, automated data collection (eg, module completion, quiz performance), and user support. A streamlined and user-friendly backend interface is essential for both researchers and educators to efficiently deliver content, monitor engagement, and provide timely feedback.

### Conclusions

This study was feasible in terms of recruitment but not retention. About half of our sample had low levels of functional disability, and in the future, recruitment via health care providers may be more appropriate, and recruitment strategies should focus on efforts to recruit individuals from heavy manual and repetitive task jobs, as they are more representative of the wider persistent LBP population. Our usability scores suggest our website meets minimal industry standards. Completion of outcomes was good, apart from ODQ. Future studies should explore culturally adapted versions of the program, developed in collaboration with patients and health care providers. Earlier and more frequent end user input may help refine language and relevance, thereby improving both recruitment and retention.

## Supplementary material

10.2196/74948 Checklist 1 CONSORT (Consolidated Standards of Reporting Trials) checklist.

## Abbreviations

CONSORT: Consolidated Standards of Reporting Trials
LBP: low back pain
MOOC: massive open online course
ODI: Oswestry Disability Index
ODQ: Oswestry Disability Questionnaire
PEI: Patient Enablement Instrument
SUS: System Usability Scale
